# Fam96a is essential for the host control of *Toxoplasma gondii* infection by fine-tuning macrophage polarization via an iron-dependent mechanism

**DOI:** 10.1371/journal.pntd.0012163

**Published:** 2024-05-07

**Authors:** Zhuanzhuan Liu, Hanying Wang, Zhiwei Zhang, Yulu Ma, Qiyue Jing, Shenghai Zhang, Jinzhi Han, Junru Chen, Yaoyao Xiang, Yanbo Kou, Yanxia Wei, Lu Wang, Yugang Wang

**Affiliations:** 1 Jiangsu Key Laboratory of Immunity and Metabolism, Department of Pathogen Biology and Immunology, Xuzhou Medical University, Xuzhou, Jiangsu Province, China; 2 Peking University Center for Human Disease Genomics, Beijing, China; 3 Department of Immunology, School of Basic Medical Sciences, Peking University, Beijing, China; 4 NHC Key Laboratory of Medical Immunology, Peking University, Beijing, China; University of Texas at El Paso, UNITED STATES

## Abstract

**Background:**

Toxoplasmosis affects a quarter of the world’s population. *Toxoplasma gondii* (*T*.*gondii*) is an intracellular parasitic protozoa. Macrophages are necessary for proliferation and spread of *T*.*gondii* by regulating immunity and metabolism. Family with sequence similarity 96A (Fam96a; formally named Ciao2a) is an evolutionarily conserved protein that is highly expressed in macrophages, but whether it play a role in control of *T*. *gondii* infection is unknown.

**Methodology/principal findings:**

In this study, we utilized myeloid cell-specific knockout mice to test its role in anti-*T*. *gondii* immunity. The results showed that myeloid cell-specific deletion of *Fam96a* led to exacerbate both acute and chronic toxoplasmosis after exposure to *T*. *gondii*. This was related to a defectively reprogrammed polarization in *Fam96a*-deficient macrophages inhibited the induction of immune effector molecules, including iNOS, by suppressing interferon/STAT1 signaling. Fam96a regulated macrophage polarization process was in part dependent on its ability to fine-tuning intracellular iron (Fe) homeostasis in response to inflammatory stimuli. In addition, Fam96a regulated the mitochondrial oxidative phosphorylation or related events that involved in control of *T*. *gondii*.

**Conclusions/significance:**

All these findings suggest that Fam96a ablation in macrophages disrupts iron homeostasis and inhibits immune effector molecules, which may aggravate both acute and chronic toxoplasmosis. It highlights that Fam96a may autonomously act as a critical gatekeeper of *T*. *gondii* control in macrophages.

## Author summary

An important feature of macrophages is their ability to adopt many different phenotypes and functions in response to extracellular or intracellular cues. Functionally well-adapted macrophages are required for the defense against *T*. *gondii*. Here, we found that Fam96a expressed in macrophages played a role in control of *T*. *gondii* infection. Mechanically speaking, Fam96a regulated intracellular iron homeostasis, which affected immune effector molecules. Our study reveals that Fam96a may autonomously act as a critical gatekeeper of intracellular parasitic control by coupling Fe metabolism to adaptative remodeling of macrophage function. It provides a novel target for prevention and treatment of toxoplasmosis and other intracellular pathogens in the future.

## Introduction

*T*. *gondii* is the pathogen that causes zoonotic toxoplasmosis [1]. Toxoplasmosis is closely related to the host immune system and affects the immunocompromised or immunodeficient host as well as immune privileged sites (brain, eye and placenta). A tightly controlled interaction between the host immune system and *T*. *gondii* is thought to be important for the control of the parasite virulence. However, many details regarding this cross-talk remain unaddressed.

Macrophages are important innate myeloid cells involved in determining the outcome of *T*. *gondii* infection. Macrophages limit toxoplasmosis through multiple mechanisms, including their intrinsic ability to limit parasite replication, their pro-inflammatory functions that promote protective NK and T-cell activities, as well as their regulatory properties that facilitate the resolution of inflammation [2]. However, *T*. *gondii* has evolved various mechanisms to subvert macrophage activation and can induce dendritic cell-like migratory properties in infected macrophages to promote dissemination [2, 3]. An important feature of macrophages is their ability to adopt many different phenotypes and functions in response to either extracellular cues or intracellular metabolic changes [4]. Iron (Fe) is a transition metal that is a key element for systemic oxygen delivery and cellular energy metabolism. Macrophages play a central role in Fe metabolism and, reciprocally, Fe trafficking, redistribution, and redox signaling properties in macrophages can regulate their polarization phenotypes and functions [5, 6].

Fam96a (also named Ciao2a) is a cytosolic protein that can regulate intracellular Fe trafficking and metabolism by (1) facilitating iron regulatory protein 1 (IRP1) acquiring iron-sulfur (Fe/S) cluster to become the cytosolic isoform of aconitase (Aco1) (2) by modulating IRP2 stabilization in a context-dependent manner [7]. Fam96a is enriched in macrophages, and *Fam96a*-deficient bone marrow-derived macrophages (BMDMs) when artificially activated by adding bacterial lipopolysaccharides (LPS) plus interferon-γ (IFN-γ) in vitro adopt an alternatively activated or anti-inflammatory (M2) phenotype instead of a classically activated or inflammatory (M1) phenotype [8], suggesting a role of Fam96a in regulating macrophage functional phenotype. However, whether Fam96a influence macrophage adaptations in response to pathogens in vivo remain largely unexplored.

Here we have explored the role of Fam96a in functional adaptations of macrophage in *T*. *gondii* infection model. We conclude that Fam96a may autonomously act as a critical gatekeeper of intracellular parasitic infection by coupling Fe metabolism to adaptative remodeling of macrophage function.

## Methods

### Ethics statement

All animal experiments were approved by the Institutional Animal Care and Use Committee of Xuzhou Medical University (Xuzhou, China, SCXK (Su) 2020–0048).

### Animals

*Lysm-cre/Fam96a*^*fl/fl*^ mice under C57BL/6J background were derived by crossbreeding *Fam96a*^*fl/fl*^ mice with *Lysm-cre* mice (both strains were provided by Dr. Lu Wang from Peking University). All mice in this study were maintained under specific pathogen-free conditions. Mice were housed in a temperature-controlled room (25 ± 2°C) and subjected to a 12-h light/dark cycle and were allowed to eat and drink *ad libitum*. A normal diet (40 ppm Fe) or a high-iron diet (5.8 ppm Fe) is based on the AIN-93G formulation (Research Diets, Beijing, China).

### T. gondii strains

Human foreskin fibroblast (HFF) cells (ATCC-SCRC-1041) were utilized to maintain cultures of *T*. *gondii* in vitro. The tachyzoites (RH strain) were added to HFF cells and incubated at 37°C and 5% CO_2_ for 72–96 h [9, 10]. The tachyzoites were collected and counted. The cysts (*T*. *gondii* Chinese 1 genotype Wh6, TgCtwh6 strain) were prepared according to a previous report [11]. The brains of infected mice with TgCtwh6 were collected, and homogenized in PBS. Number of cysts was counted and then used to challenge mice for further infection experiments.

### Macrophage isolation and culture

Bone marrow-derived macrophages (BMDMs) were obtained by differentiation of bone marrow cells in the presence of macrophage colony-stimulating factor (M-CSF) according to a previous report [12]. Briefly, the femur and tibia were isolated from *Fam96a*^*fl/fl*^ and *Lysm-cre*/*Fam96a*^*fl/fl*^ mice. After cutting the epiphyses of the bones, the bone marrow cavity was flushed using PBS. The precipitation was collected and lysed using red blood cell lysis buffer. The cells were diluted in medium [DMEM containing 10% fetal bovine serum (FBS), 20 ng/ml M-CSF] and cultured at 37°C with 5% CO_2_. On the sixth day, BMDMs were collected by 0.25% trypsin treatment and seeded in 6-well or 12-well plate for further experiments.

Peritoneal macrophages (PEMs) were collected from *Fam96a*^*fl/fl*^ and *Lysm-cre/Fam96a*^*fl/fl*^ mice after infection with *T*. *gondii* RH tachyzoites [13]. The mice were euthanized and then peritoneal lavage (5 mL of cold D-Hank’s solution) was collected from the peritoneal cavity. After centrifugation at 300 *g* for 10 min, the pellets were resuspended in RPMI1640 medium for 3 h at 37°C. The adherent cells were PEMs.

### Infection challenges

*Fam96a*^*fl/fl*^ and *Lysm-cre*/*Fam96a*^*fl/fl*^ mice were intraperitoneally injected with RH tachyzoites (1000 per mouse) or orally ingested with TgCtwh6 cysts (50 per mouse). The survival of mice was monitored daily. In vitro, RH tachyzoites were incubated with BMDMs or HFF cells at a ratio of 2:1. The uninvaded tachyzoites were excluded 30 min after incubation by 3 times washing.

### Quantitative polymerase chain reaction (qPCR)

Total RNAs from BMDMs or PEMs were isolated and purified using TRIzol reagent (Invitrogen, Carlsbad, CA, USA). The RNA amount and purity of each sample were quantified using NanoDrop ND-1000 (NanoDrop, Wilmington, DE, USA). cDNA was prepared by reverse transcription using the cDNA synthesis kit (Yasen, Shanghai, China).

DNA of tissues and cells were extracted using the DNA extraction kit (Tiangen, Beijing, China). qPCR was performed using SYBR Green Master Mix (Yasen, Shanghai, China) on a Roche LightCycler 480. *T*. *gondii* surface antigen 1 (SAG1) gene was used to determine the relative parasite load. Data was analyzed by comparing the threshold cycle (Ct) value of the target gene to *β-actin* using the formula 2^-ΔΔCt^. Primer sequences are listed in S1 Table.

### Western blot analysis

The following primary antibodies were used for immunoblot: anti-FTH1 (cat no., 3998S), anti-IRP1(cat no., 20272S), anti-STAT1(cat no., 14994) and anti-pSTAT1(Tyr701) (cat no., 9167) were all purchased from Cell Signaling Technology (Danvers, MA, USA); anti-IRP2 (cat no., NB100-1798), and anti-FPN (cat no., NBP1-21502) were from Novus Biologicals (Centennial, CO, USA); anti-iNOS (cat no., ab178945) were from abcam (Cambridge, MA, USA); anti-Arg1 (cat no., 16001-1-AP) were from Proteintech (Wuhan, China).

### Quenchable iron pool (QIP) assay

We followed a previously described protocol [14]. In brief, BMDMs were stained with 1 μM Calcein-AM (425201, BioLegend, San Diego, CA, USA) for 15 min at 37°C, and then treated with PBS or FeHQ solution (5 μM FeCl_2_ and 10 μM 8-hydroxyquinoline) for 30 min at 37°C. The MFI of Calcein staining was determined by Flow cytometry. The QIP per sample was calculated as MFI(PBS) minus MFI(FeHQ).

### Oxygen consumption rate (OCR) and extracellular acidification rate (ECAR) measurements

OCR and ECAR of BMDMs were measured using a Seahorse XFe24 Analyzer (Agilent Technologies, Santa Clara, CA, United States) [15]. BMDMs (80,000 cells/well) were plated into an XF24-well. After measuring the basal respiration, OCR analysis was performed by sequential injection of 1 μM oligomycin, 1 μM carbonyl cyanide *p*-(trifluoromethoxy) phenylhydrazone (FCCP), and 1 μM rotenone/antimycin (Rot A). For ECAR assay, BMDMs were sequentially added with 100 mM glucose, 1 μM oligomycin, and 500 mM 2-Deoxy-D-glucose (2-DG). The OCR and ECAR values were normalized with protein concentration determined by the Bradford method.

### Cytokines analysis

The blood samples were collected from infected *Fam96a*^*fl/fl*^ and *Lysm-cre*/*Fam96a*^*fl/fl*^ mice at day 4, 7 post infection. The levels of TNF-α and IFN-γ in serum were detected using Cytometric Bead Array (CBA) kit (cat no.,552364, USA). The assay was carried out following the manufacturer’s recommended protocol. The results were analyzed using FCAP software.

### Immunofluorescence microscopy

BMDMs or HFFs were challenged by *T*. *gondii* tachyzoites (MOI = 2) for 12 h and were fixed with 4% paraformaldehyde. Cells were permeabilized with 0.5% TritonX-100 in PBS. After blocking with 2% BSA, cells were incubated with anti-SAG1 (cat no., AM316809U-N) for 16–24 h, and stained with Coralite594-conjugated goat anti-mouse IgG(H+L) (cat no., SA00013-3) for 1 h and DAPI. Images were acquired on Olympus IX73 microscopy.

### Hematoxylin-eosin (HE) staining

The tissue sections of lungs from infected mice were fixed in 4% paraformaldehyde solution, and then embedded in paraffin. Sections of 5–8 μm were taken and stained with Hematoxylin and Eosin. The images were collected using an optical microscope.

### Giemsa staining

The abdominal cavity of each *Fam96a*^*fl/fl*^ or *Lysm-cre*/*Fam96a*^*fl/fl*^ mice infected with *T*. *gondii* tachyzoites was washed with 2 mL PBS. The peritoneal fluid was collected, and equal amounts of peritoneal fluid was evenly smeared on the glass slide. It was fixed in methanol and stained with Giemsa for 20 min. The images were collected using an optical microscope.

### Enzyme-linked Immunosorbent Assay (ELISA)

Cell culture supernatant was collected from BMDMs that were stimulated with LPS or *T*. *gondii* tachyzoite for 12 h. Then, the supernatant (100 μL) was added to an ELISA plate well that was pre-coated with anti-TNFα or IL-6 primary antibody, and incubated at 25°C for 2 h. The plate wells were then washed for 3 times, a biotin-labeled anti-TNFα or -IL-6 secondary antibody was added and incubated at 25°C for 1 h. After washing again for 3 times, HRP-labelled avidin was added and incubated at 25°C for 30 min, and the plate wells were washed again. Finally, tetramethylbenzidine (TMB) substrate solution was added and incubated for 15 min. The reaction was stopped with a stop solution and the absorbance values at 450 nm and 570 nm were recorded using a microplate spectrophotometer.

### Measurement of NO production

The concentration of NO released in the supernatant from the cultured BMDMs was detected using a Griess Reagent Kit for Nitrite Determination (Invitrogen, Carlsbad, CA, USA). An equal volume of N-(1-naphthyl) ethylenediamine and sulfanilic acid were mixed to form the Griess Reagent. The supernatant collected from the BMDMs was mixed with the Griess Reagent and incubated for 30 min at room temperature. The absorbance at 548 nm was measured using a microplate spectrophotometric reader.

### Determination of Arginase activity

Arginase activity in BMDMs was determined using an arginase activity assay kit (Solarbio, Beijing, China). The samples were collected and extracted by ultrasonic disruption. The supernatant was collected after centrifugation at 12,000×*g* at 4°C for 20 min, and detected according to the manufacturer’s instructions. Standard curve was generated by serial dilution of urea. Measurement was performed at 560 nm in a spectrophotometer.

### Statistical analysis

The statistical analysis of data was conducted by SPSS 19.0 software. Statistical significance was determined using the unpaired two-tailed Student’s t-test for single variables and one- or two-way ANOVA followed by Bonferroni post tests for multiple variables. Geha-Breslow-Wilcoxon test was used for survival comparison. A value of *P*<0.05 was considered statistically significant.

## Results

### Fam96a is required for optimal pro-inflammatory responses in macrophages

**Fig 1:**
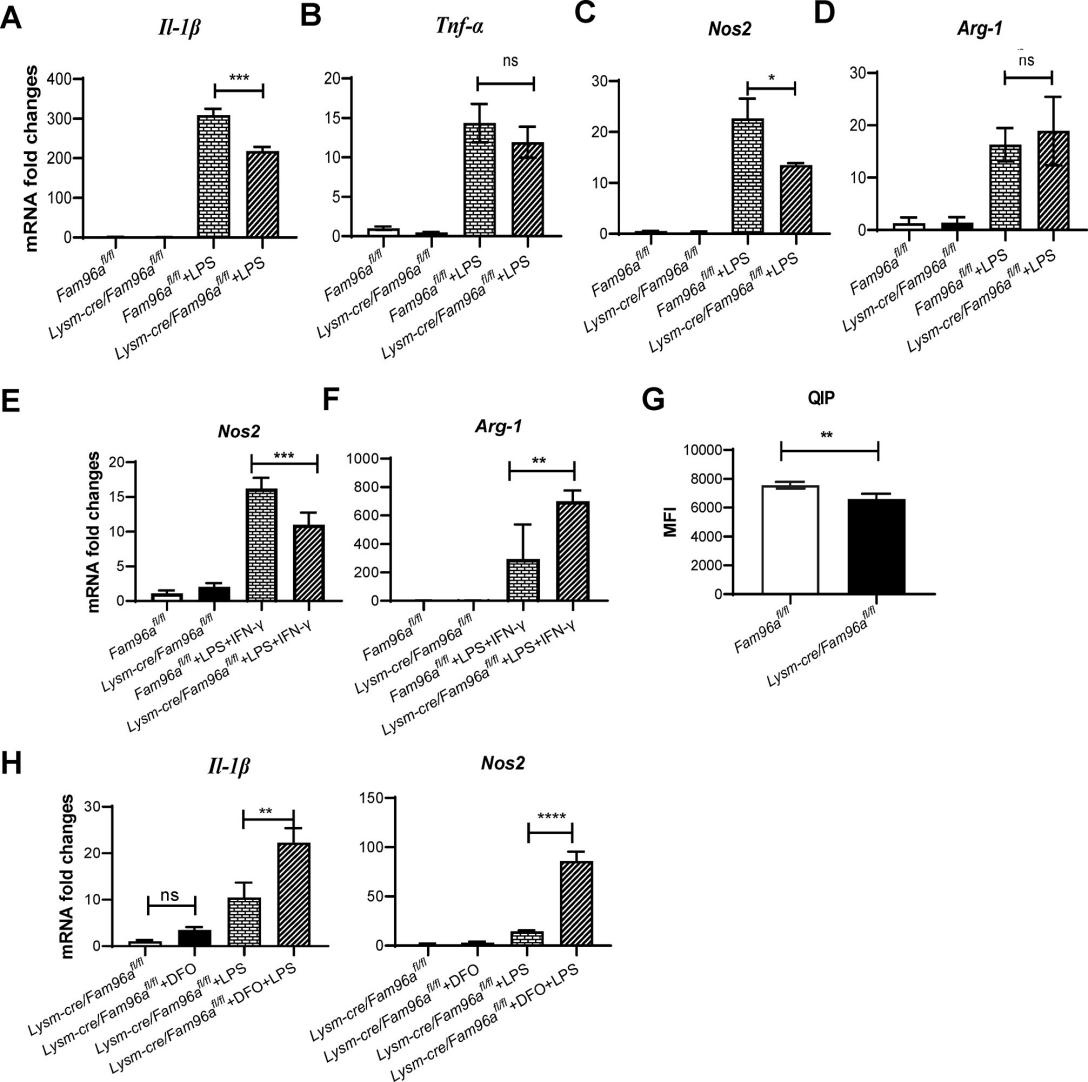
Fam96a is required for optimal inflammatory responses in macrophages by influencing intracellular Fe levels. (A-D) BMDMs derived from *Fam96a*^*fl/fl*^ and *Lysm-cre*/*Fam96a*^*fl/fl*^ mice were stimulated with LPS (100 ng/mL) for 12 h in vitro. The mRNA expression levels of (A) *Il-1β*, (B) *Tnf-α*, (C) *Nos2*, and (D) *Arg-1* were evaluated by RT-qPCR. (E and F) BMDMs were stimulated with LPS (100 ng/mL) +IFN-γ (100 ng/mL) for 12 h, and then the mRNA expression levels of (E) *Nos2* and (F) *Arg-1* were determined by RT-qPCR. The mRNA expression levels are normalized according to their corresponding *β-actin* mRNA expression levels. (G) Quenchable iron pools (QIPs) in wild-type (derived from *Fam96a*^*fl/fl*^ mice) and *Fam96a*-deficient (derived from *Lysm-cre*/*Fam96a*^*fl/fl*^ mice) BMDMs. n = 4. (H) *Fam96a*-deficient BMDMs were treated with either DFO (200 μM) for 12h, or LPS (100 ng/mL) for 12 h, or DFO for 12 h and then LPS for another 12 h. The mRNA expression levels of *Il-1β* and *Nos2* were then determined by RT-qPCR. n = 3. Statistical analysis with one-way ANOVA (A-F, H) and t-test (G). *P < 0.05; ***P < 0.001.

To investigate the possible roles of Fam96a in macrophages, we compared the pro-inflammatory responses of BMDMs originated from *Fam96a*^*fl/fl*^ and *Lysm-cre*/*Fam96a*^*fl/fl*^ mice upon LPS stimulation in vitro. The results indicated that the relative gene expressions of interleukin (*Il*)*-1β* and inducible nitric oxide synthase (*Nos2*, gene encoding for iNOS protein) were reduced in *Fam96a*-deficient macrophages after 12 h of LPS stimulation compared to those in the wild-type controls (Fig 1A and 1B). However, the induction of tumor necrosis factor (*Tnf-α*) and arginase (*Arg-1*) were no significant difference 12 h post LPS stimulation (Fig 1C and 1D). Furthermore, the protein levels of TNFα and IL-6 released in the supernatant were downregulated in *Fam96a*-deficient BMDMs 12 h post LPS or *T*. *gondii* tachyzoite RH stimulation (S1A and S1B Fig). The amount of NO released was reduced, while the arginase activities in the cells remained the same (S1C and S1D Fig). Under M1 cell polarization condition (i.e., LPS plus IFNγ), *Fam96a*-deficient BMDMs were relatively less effective in promoting *Nos2* expression, but more effective in inducing *Arg-1* expression (Fig 1E and 1F). In addition, the relative gene expressions of *Il-1β*, *Tnf-α*, and *Il-6* were also reduced in *Fam96a*-deficient peritoneal macrophages after 4 h of LPS or *T*. *gondii* tachyzoite RH stimulation in vitro (S2 Fig). The data collectively suggests that macrophage intracellular Fam96a is required for optimal phenotypic polarization in response to inflammatory stimuli.

Fam96a is thought to be a cytoplasmic Fe-S clusters assembly protein potentially involved in regulating intracellular Fe homeostasis [7, 16]. We therefore checked whether macrophage Fam96a-deficieny can affect their Fe pools. We measured the quenchable iron pools (QIPs), which is inversely related to the cytoplasmic labile iron pools (LIPs)[14]. *Fam96a*-deficient BMDMs had relatively smaller QIPs (Fig 1G), indicating that *Fam96a*-deficient BMDMs had increased LIPs and therefore more cytosolic labile Fe.

To check whether the increased LIPs in *Fam96a*-deficient BMDMs is responsible for disturbing cellular pro-inflammatory responses, we treated *Fam96a*-deficient BMDMs with the iron chelator deferoxamine (DFO) for 12 h to reduce cellular LIPs before LPS stimulation. DFO-treated cells had increased expression of *Il-1β* and *Nos2* upon stimulation with LPS (Fig 1H), implicating that tightly regulated cytosolic labile Fe pools by Fam96a is required for macrophage to engage an optimal inflammatory response after sensing an inflammatory stimuli.

### BMDM intrinsic Fam96a is critical for resistance to *T*. *gondii* infection

**Fig 2:**
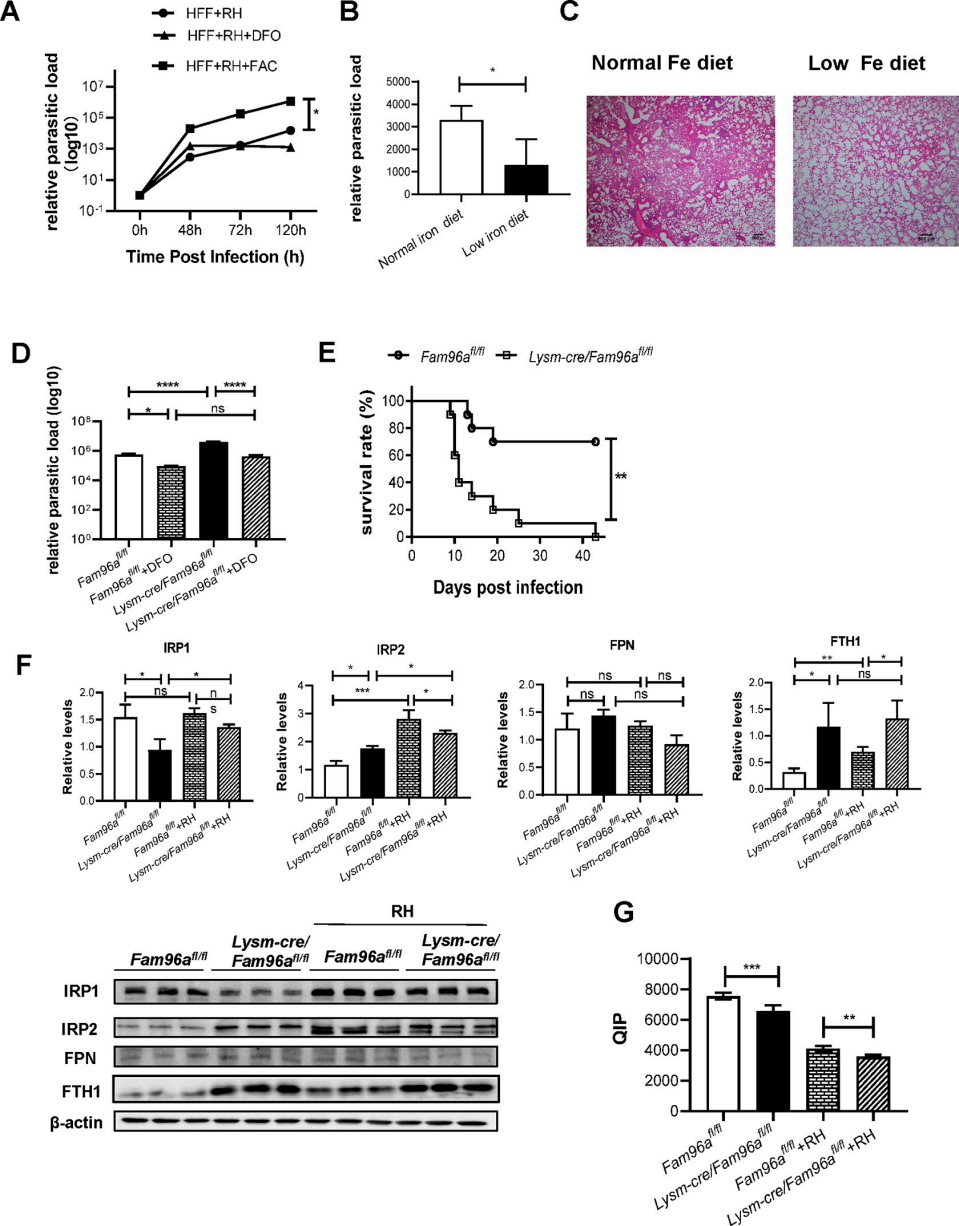
BMDM intrinsic Fam96a is critical for resistance to *T*. *gondii* infection. (A) 2×10^5^ HFF cells were pre-treated with medium, DFO or FAC (50 μM) for 12 h, and then infected with *T*. *gondii* tachyzoite RH (MOI = 2). The cells were collected at 0 h, 48 h, 72 h, and 120 h. The parasitic loads were detected by qPCR. n = 3. (B) *Fam96a*^*fl/fl*^ mice were fed on a normal or low iron diet, and then orally infected with *T*. *gondii* cysts (50 per mouse). The relative parasitic load in brain was determined 4 weeks after infection. n = 5/group. (C) Hematoxylin-eosin staining of lung tissue sections collected from the indicated mice 4 weeks after infection. n = 5/group. (D) BMDMs derived from *Fam96a*^*fl/fl*^ and *Lysm-cre*/*Fam96a*^*fl/fl*^ were pre-treated with medium or DFO for 12 h, and then infected with *T*. *gondii* tachyzoites. The parasite loads 24 h after infection were determined by qPCR. n = 3. (E) Survival rates of *Fam96a*^*fl/fl*^ and *Lysm-cre*/*Fam96a*^*fl/fl*^ mice after orally infected with *T*. *gondii* cysts (50 per mouse). n = 5/group. (F) Immunoblot analysis and densitometry quantification of the indicated proteins that involve in regulating intracellular iron pools in BMDMs before and after 12 h *T*. *gondii* tachyzoite RH infection. n = 3. (G) QIPs in the indicated BMDMs before and after 12h post infection. n = 4. The QIPs data before infection are adopted from Fig 1G. Data are represented as mean ± SD. Statistical analysis with a two-tailed unpaired t-test (B), one-way ANOVA (D, F, and G), log rank test (E), and two-way ANOVA analysis (A). *P < 0.05; **P< 0.01; ***P < 0.001; ****p < 0.0001; ns, no statistical significance.

Iron is essential for the survival of intracellular parasites, including *Toxoplasma gondii* [17]. We first tested the role of Fe in regulating *T*. *gondii* proliferation in HFF cells by artificially disturbing intracellular LIPs in vitro. Adding Ferric ammonium citrate (FAC) in the cell culture medium promoted *T*. *gondii* tachyzoites (RH strain) proliferation, while chelating Fe with DFO had a trend to reduce *T*. *gondii* proliferation but it did not reach statistical significant yet (Fig 2A). Next, we fed wild-type (i.e., *Fam96a*^*fl/fl*^) mice with a low iron diet and then infected them with TgCtwh6 cysts (the Chinese 1 genotype Wh6 strain). Mice on the low iron diet had significantly reduced parasite loads in brain 4 weeks after infection (Fig 2B), and the parasite infection-induced lung inflammation was also diminished (Fig 2C). Thus, the cellular iron status is closely associated with toxoplasmosis.

Since our data indicate Fe is involved in Fam96a-regulated macrophage pro-inflammatory responses in vitro, we therefore treated WT or *Fam96a*-deficient BMDMs with DFO for 12 h, and then infected the cells with *T*. *gondii* RH tachyzoites. The parasite loads were increased in *Fam96a*-deficient BMDMs compared to WT controls, and DFO treatment was effective to ameliorate parasite overload in Fam96a-deficint cells (Fig 2D), indicating *Fam96a*-regulated Fe homeostasis is critical for resisting *T*. *gondii* infection. To further approve this in vivo, we orally infected *Fam96a*^*fl/fl*^ and *Lysm-cre*/*Fam96a*^*fl/fl*^ mice with TgCtwh6 cysts (50 per mouse). Fam96a-deficient mice were more sensitive to TgCtwh6-induced chronic infection and had reduced survival rate over time post infection (Fig 2E).

Further, we found *T*. *gondii* RH infection can induce a cellular Fe response in WT BMDMs, indicated by increased expressions of iron-regulatory protein IRP2 and FTH1 and no significant differences for IRP1 and FPN 12 h post infection (Fig 2F). Fam96a-deficiency disturbed this cellular Fe response. Although the basal levels of IRP2 and FTH1 were increased in *Fam96a*-deficient BMDMs compared with the WT control, they failed to increase further after infection (Fig 2F). Intracellular QIP values were reduced after *T*.*gondii* infection, and Fam96a deficiency further reduced QIPs, suggesting more freely available Fe presented in *Fam96a*-deficient BMDMs post *T*. *gondii* RH infection (Fig 2G). Together, the data suggest that Fam96a-regulated iron homeostasis is critical for optimal functional adaptations of BMDMs in fighting against *T*. *gondii* infection.

### Fam96a regulates *T*. *gondii* infection-induced iNOS expression in macrophages

**Fig 3:**
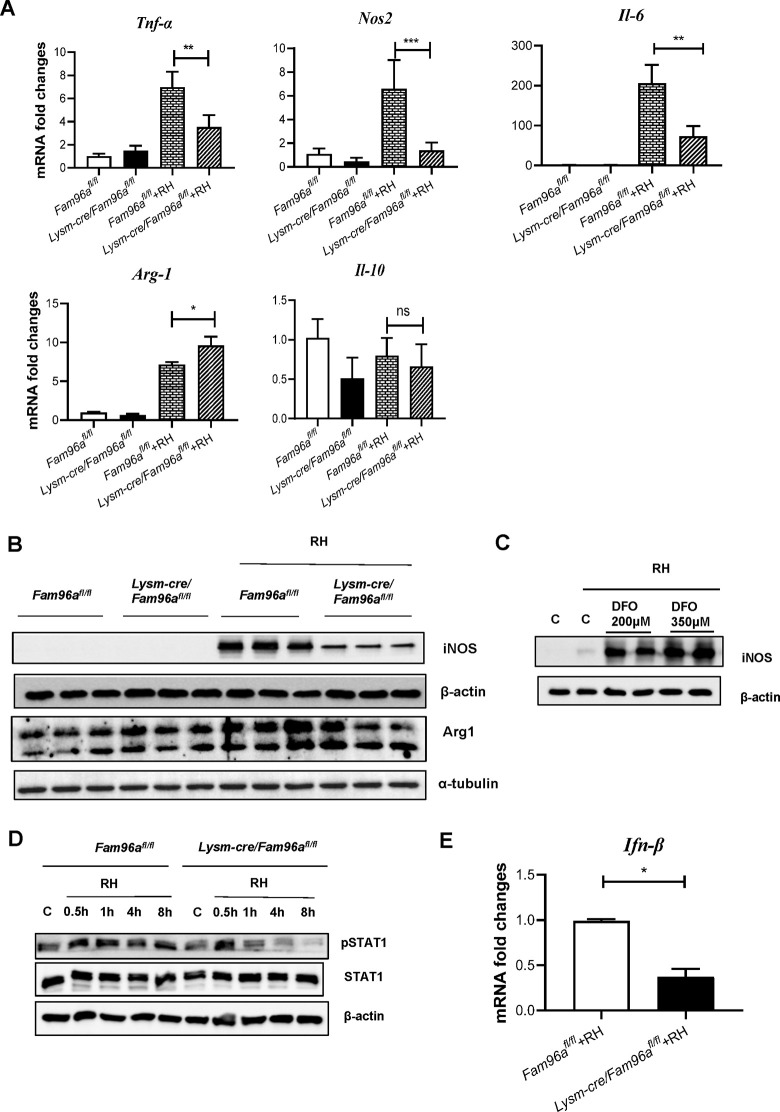
Macrophage-selective Fam96a ablation inhibits iNOS expression. (A) BMDMs originated from *Fam96a*^*fl/fl*^ and *Lysm-cre*/*Fam96a*^*fl/fl*^ mice were infected with *T*. *gondii* RH tachyzoites for 12 h. The mRNA levels of *Tnf-α*, *iNos*, *Il-6*, *Arg-1*, and *Il-10* were determined by RT-qPCR. n = 3. (B) Immunoblot analysis of iNOS and Arg-1 expression in the indicated BMDMs before or after 12 h RH infection. n = 3. (C) BMDMs derived from *Fam96a*^*fl/fl*^ mice were incubated with vehicle control (C for short) or DFO(200 μM or 350 μM)for 12 h, and then infected with *T*. *gondii* RH tachyzoites for 12 h. The expression of iNOS was determined by immunoblot. (D) Immunoblot analysis of RH infection-induced STAT1 phosphorylation in BMDMs originated from *Fam96a*^*fl/fl*^ and *Lysm-cre*/*Fam96a*^*fl/fl*^ mice. (E) The mRNA expression levels of *Ifn-β* 30 min post infection were determined by RT-qPCR. n = 3. Data are represented as mean ± SD. Statistical analysis with one-way ANOVA analysis (A) and a two-tailed unpaired t-test (E). *P < 0.05; **P< 0.01; ***P< 0.001; ns, no statistical significance.

The ability of macrophage to control the intracellular parasite *T*. *gondii* depends on a balance between inflammatory and anti-inflammatory gene expressions. Compared to the controls, the levels of *Tnf-α*, *Nos2*, and *Il-6* induced by *T*. *gondii* infection were reduced in *Fam96a*-deficient BMDMs, but the *Arg-1* expression levels were instead increased and the *Il-10* expression levels were normal (Fig 3A). Notably, the iNOS protein expression induced by *T*. *gondii* infection was inhibited in *Fam96a*-deficient macrophages (Fig 3B). This is related to Fe-related events, as DFO treatment significantly increased iNOS expression in WT BMDMs after *T*. *gondii* infection (Fig 3C).

Signal transducer and activator of transcription (STAT) factors regulate antimicrobial defense and inflammatory response in host [18]. Phosphorylation of STAT1 induced by type I or II interferon (IFN-I or IFN-II) signaling regulates the expression of *Nos2*, which is important for host control of *T*. *gondii* infection [19, 20]. We found that *T*. *gondii-*infection-induced STAT1 phosphorylation declined faster in *Fam96a*-deficient BMDMs than that in the WT controls (Fig 3D), suggesting Fam96a may be involved in regulating IFN signaling. In agreement with this, *T*. *gondii* infection-induced less *Ifn-β* expression in Fam96a-deficeint BMDMs (Fig 3E). Together, the findings suggest that macrophage Fam96a may regulate iNOS expression via influencing IFN-I/STAT1 signals.

### Fam96a influences macrophage metabolic remodeling in vitro

**Fig 4:**
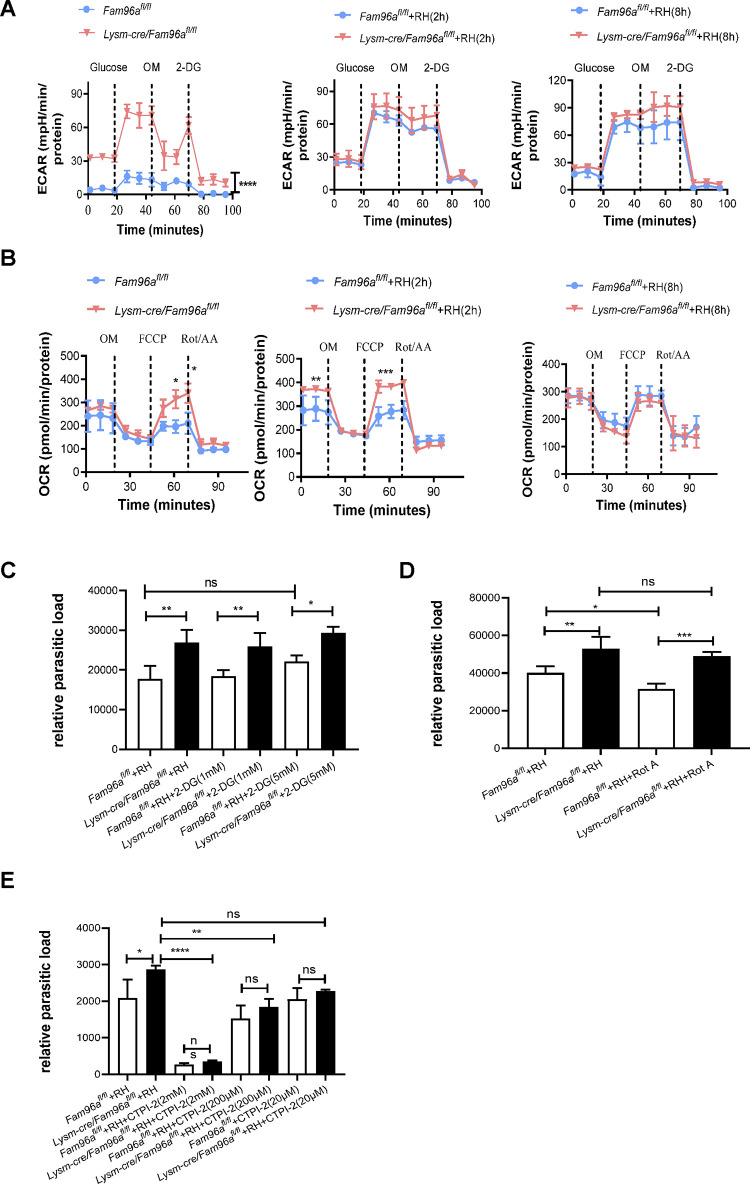
Fam96a influences macrophage metabolic remodeling in vitro. (A) Extracellular acidification rate (ECAR) of BMDMs before and after 2 h or 8 h RH tachyzoites infection. n = 3. (B) Oxygen consumption rate (OCR) of BMDMs before and after 2 h or 8 h RH tachyzoites infection. n = 3. (C) BMDMs were treated with 2-DG (1 mM or 5 mM), and then infected with RH tachyzoites. The parasite loads 12 h post infection were then determined. n = 3. (D) The parasite loads in BMDCs with or without Rot/AA (0.5 μM) treatment. n = 3. (E) The parasite loads in BMDCs with or without CTPI-2 (2mM, 200 μM, and 20 μM) treatment. n = 3. OM, Oligomycin; FCCP, carbonyl cyanide 4-(trifluoromethoxy)phenylhydrazone; Rot/AA, Rotenone/Antimycin A; 2-DG, 2-Deoxy-D-glucose. Statistical analysis with one-way (C-F) and two-way (A and B) ANOVA. *P< 0.05; **P < 0.01; ***P< 0.001.

In response to proinflammatory stimuli, macrophages undergo profound metabolic remodeling to support not only the biosynthetic and bioenergetic requirements of the cell but also dictate the inflammatory response [21]. To explore whether Fam96a regulating macrophage functional adaptation requires metabolic remodeling, we first measured the extracellular acidification rate (ECAR) and the real-time oxygen consumption rate (OCR) of WT and *Fam96a*-deficient BMDMs before and after *T*. *gondii* RH infection. At the basal condition, *Fam96a*-deficient BMDMs exhibited a relatively higher glycolytic rate and a higher maximal respiratory rate (i.e., tested upon the mitochondrial uncoupler FCCP challenge) when compared to WT BMDMs (Fig 4A and 4B), indicating Fam96a has a role in control of basal metabolic state. After infection with *T*. *gondii*, the ECAR levels were increased in WT (the Warburg effect) but not in *Fam96a*-deficient BMDMs (Fig 4A, and the merged graph in S3A Fig). In addition, the basal and maximal respiratory rates in *Fam96a*-deficient BMDMs were transiently increased at 2 h post infection and they became comparable to the control at 8 h post infection (Fig 4B, and the merged graph in S3B Fig). The data suggest that Fam96a also has a role in control of metabolic reprograming after the parasite stimulation.

Next, we used 2-Deoxy-d-glucose (2-DG), a glycolysis inhibitor via inhibiting hexokinase [22], to treat BMDMs right before RH infection. 2-DG treatment had no impact on *T*. *gondii* infection efficiency (Fig 4C), suggesting that glycolysis *per se* may not be relevant for macrophage control of *T*. *gondii*. We then used rotenone/antimycin A (Rot/AA), which is the mitochondrial electron transport chain complex I/III inhibitor and can promote reactive oxygen species (ROS) production, to treat the cells right before RH infection [23, 24], and the treatment reduced *T*. *gondii* load in WT BMDMs but not in *Fam96a*-deficient BMDMs (Fig 4D), suggesting that Fam96a may regulate the mitochondrial oxidative phosphorylation or related events that involved in control of *T*. *gondii*.

Fam96a controls the cytoplasmic aconitase (Aco1) enzyme activity via Fe-S transfer, and *Fam96a*-deficient cells lose Aco1 activity and therefore are more likely to accumulate cytosolic citrate. Thus, we used the mitochondrial citrate carrier Slc25a1 inhibitor CTPI-2 [25, 26] to block mitochondrial citrate transferring to the cytosol right before RH infection. When incubated with CTPI-2, the number of *T*. *gondii* was reduced in both WT and Fam96a-deficeint BMDMs (Fig 4E), suggesting that Slc25a1 is an important node for the parasite control. However, whether this is related to cytosolic citrate requires further investigation.

### Fam96a regulates macrophage functional adaptation in vivo

**Fig 5:**
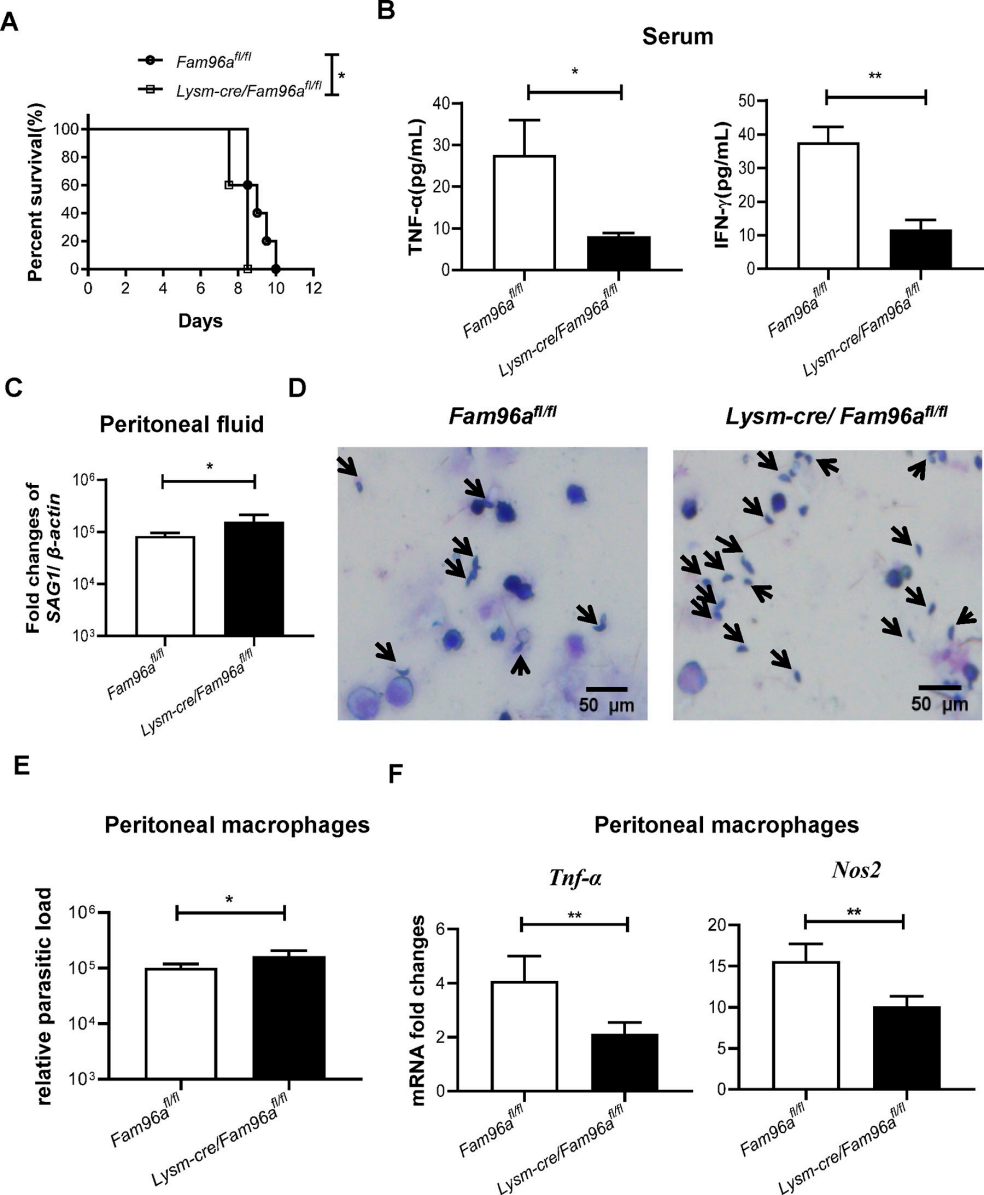
Fam96a regulates macrophage functional adaptation in vivo. *Fam96a*^*fl/fl*^ and *Lysm-cre*/*Fam96a*^*fl/fl*^ mice were intraperitoneally injected with RH tachyzoites (10^3^ per mouse). (A) The survival curves post infection. n = 5. (B) At the fourth day post infection, serum TNF-ɑ and IFN-γ levels were determined. n = 5. (C) The parasite loads in peritoneal fluid 4 days post infection were analyzed using qPCR. n = 4. (D) Giemsa staining of *T*. *gondii* tachyzoites in the peritoneal fluid collected from the indicated mice 4 days post infection. The black arrows indicate the tachyzoites. Scale bar = 50 μm. (E) The parasite loads in peritoneal macrophages isolated from the indicated mice 4 days post infection. n = 5. (F) The mRNA levels of *Tnf-α* and *Nos2* in peritoneal macrophages isolated from the indicated mice 6 days post infection. n = 4. Data are represented as mean ± SD. Statistical analysis with log rank test (A) or a two-tailed unpaired t-test (B and C, E and F). *P< 0.05; **P< 0.01.

To investigate whether Fam96a influences macrophage functional adaptation in vivo, we first challenged *Fam96a*^*fl/fl*^ and *Lysm-cre*/*Fam96a*^*fl/fl*^ mice with *T*. *gondii* RH tachyzoites. *Fam96a*^*fl/fl*^ mice died between 7.5 and 8.5 days, whereas *Lysm-cre*/*Fam96a*^*fl/fl*^ mice died between 8.5 and 10 days. Log-rank test showed that Fam96a ablation in myeloid cells shortened survival time after intraperitoneally challenged with *T*.*gondii* RH tachyzoites (χ^2^ = 4.77, *P* < 0.05, Fig 5A). The serum levels of TNFα and IFN-γ were relatively lower in *Lysm-cre*/*Fam96a*^*fl/fl*^ mice compared to the control group 4 days post-infection (Fig 5B). The parasite loads in both peritoneal fluid and peritoneal macrophages were increased in *Lysm-cre*/*Fam96a*^*fl/fl*^ group compared to the control group (Fig 5C–5E). In line with our in vitro findings (Fig 3A), peritoneal macrophages collected from *Lysm-cre*/*Fam96a*^*fl/fl*^ mice 6 days post-infection expressed relatively lower levels of *Tnf-α* and *Nos2* compared to the controls (Fig 5F). These findings support that Fam96a is intrinsically critical to regulate macrophage functional adaptation in vivo.

## Discussion

Multiple lines of experimental findings described in this study provide the genetic evidence for the involvement of Fam96a, a protein involved in regulating intracellular Fe homeostasis, in the regulation of immune response against *T*. *gondii* infection in vivo. Specifically, Fam96a regulates macrophage-mediated immune responses by fine-tuning adaptative remodeling of macrophage when facing the parasite infection. How Fam96a controls immune response is intriguing. We found that Fe is one of the contributing factors. In our studies, iron chelator DFO could reverse the functional states of BMDMs. Other studies have found that macrophage mitochondrial iron levels can even determine systemic metabolic homeostasis in mice [27]. Over the last decades, much attention has been paid to the Krebs cycle as the central immunometabolic hub of the macrophage [28], our study has reinforced the importance of Fe metabolism in macrophage polarization and defense against at least intracellular pathogen such as *T*. *gondii*, and suggests that targeting Fe pathways may be important for protecting against toxoplasmosis.

Iron is essential for both the host and the parasite. It can work as a cofactor in enzymes of respiration and replication, and it can also react with H_2_O_2_ to form highly reactive hydroxyl and hydroperoxyl radicals which can cause extensive damage to the cells. Tightly controlled cellular Fe distribution is important for life. Type I interferon response mediated imbalance of macrophage iron homeostasis could promote *Candida glabrata* fitness and immune evasion [29]. Our study suggest Fam96a acts as a very important node to fine-tune intracellular Fe, and *Fam96a*-deficient macrophages have increased LIPs. *T*. *gondii* replication is Fe-dependent [30], increased LIPs are therefore beneficial for *T*. *gondii* infection.

Fam96a regulates cellular Fe homeostasis via influencing IRP1/2 [7]. The IRP1/2 system is responsible for directly regulating cellular Fe homeostasis [31]. We found that Fam96a may regulate IRP1/2 differently in different cell types. In brown adipocytes, the intracellular LIPs of *Fam96a*^*-/-*^ adipocytes was reduced, coupled with the higher expression of IRP1 [16]. In contrast, *Fam96a*^*-/-*^ BMDMs had increased LIPs, decreased IRP1 and increased IRP2. Therefore, the molecular interconnection network of Fam96a in different cell types may require further investigation.

In addition, *Fam96a*-deficient macrophages inhibited the induction of immune effector molecules, including iNOS. iNOS-catalyzed production of NO can effectively kill pathogens [32]. Thus, Fam96a may control *T*. *gondii* infection by fine tuning immune effector functions. We found that Fam96a can influence STAT1 signaling. STAT1 signaling is responsible for the production of iNOS [33]. It is known that IRP1 inhibits the expression of 5’-aminolevulinate synthase 2 [34], which is a key enzyme in heme synthesis, and heme can negatively regulate the STAT1 pathway [35]. Whether Fam96a influences STAT1 signaling via the IRP1-heme axis requires future investigation.

*T*. *gondii* infection causes metabolic reprogramming of bone marrow derived dendritic cells(BMDCs), including upregulated glycolysis, altered tricarboxylic acid(TCA) cycle and a decrease in oxidative phosphorylation, which is thought to facilitate phagocytosis, antigen processing and cytokine production [36]. However, our data indicated that although *Fam96a*-deficient BMDMs had greatly increased glycolytic ability, inhibiting glycolysis by 2-DG had no impact on *T*. *gondii* infection efficiency, suggesting that increasing glycolysis *per se* may be irrelevant for the control of *T*. *gondii* in macrophage. Instead, our data suggests that mitochondrial oxidative phosphorylation or its related events may rather be relatively more important. In addition, our data suggests that cytosolic citrate levels maybe important for the control of *T*. *gondii* infection. However, the underlying mechanism remains unknown.

It should be mentioned that in addition to macrophages, some other types of myeloid cells are also *Fam96a*-deficient in *Lysm-cre/Fam96a*^*fl/fl*^ mice. Whether Fam96a in other myeloid cells, such as neutrophils, is involved in the control of *T*. *gondii* infection requires future exploration.

In conclusion, our study reveals that Fam96a may autonomously act as a critical gatekeeper of intracellular parasitic control in macrophages by coupling Fe metabolism to adaptative functional remodeling. The molecular mechanisms provided in here may help to find novel targets for prevention and treatment of toxoplasmosis and other intracellular pathogens in the future.

## Supporting information

S1 Table The PCR primer sequences used in this study. (XLSX)

S1 Fig Fam96a is required for optimal proinflammatory responses in BMDMs. BMDMs isolated from *Fam96a*^*fl/fl*^ and *Lysm-cre*/*Fam96a*^*fl/fl*^ mice were stimulated with either LPS (100 ng/mL), LPS plus IFNγ (100 ng/mL), or *T*. *gondii* tachyzoite RH (MOI = 2) for 12h, the supernatant was then collected. The concentrations of *TNFα* (A), *Il-6* (B), and NO (C) in the supernatant were then measured. The arginase activities (D) in the cell lysate were also monitored. *P < 0.05; **P < 0.01; ***P < 0.001; ****P < 0.0001; ns, no statistical significance.
(TIF)

S2 Fig Fam96a is required for optimal proinflammatory responses in peritoneal macrophages. Peritoneal macrophages isolated from *Fam96a*^*fl/fl*^ and *Lysm-cre*/*Fam96a*^*fl/fl*^ mice were stimulated with LPS (100 ng/mL) or *T*. *gondii* tachyzoite RH (MOI = 2) for 4 h in vitro. The mRNA expression levels of (A) *Il-1β*, (B) *Tnf-α*, and (C) *Il-6* were then evaluated by qRT-PCR. **P < 0.01; ***P < 0.001;
(TIF)

S3 Fig Fam96a influences macrophage metabolic remodeling in vitro. (A) the three individual ECAR graphs seen in the main Fig 4A were merged in this graph. (B) the three individual OCR graphs seen in the main Fig 4B were merged in this graph.
(TIF)
