# Supplementary figures and images for: Fam96a is essential for the host control of Toxoplasma gondii infection by fine-tuning macrophage polarization via an iron-dependent mechanism
Source: PLoS Negl Trop Dis. 2024 May 7;18(5):e0012163. doi: 10.1371/journal.pntd.0012163 (PMC11101080; doi:10.1371/journal.pntd.0012163)

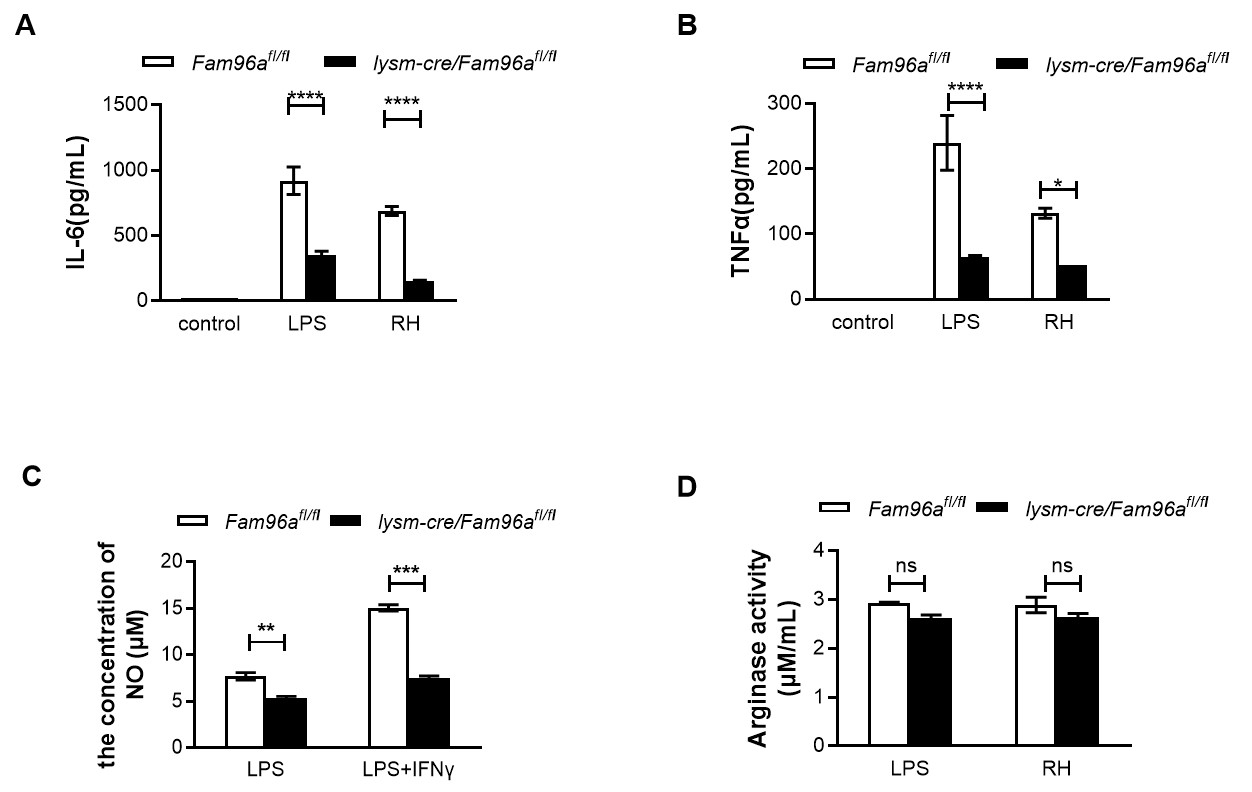

Supplement: S1 Fig — BMDMs isolated from Fam96afl/fl and Lysm-cre/Fam96afl/fl mice were stimulated with either LPS (100 ng/mL), LPS plus IFNγ (100 ng/mL), or T. gondii tachyzoite RH (MOI = 2) for 12h, the supernatant was then collected. The concentrations of TNFα (A), Il-6 (B), and NO (C) in the supernatant were then measured. The arginase activities (D) in the cell lysate were also monitored. *P < 0.05; **P < 0.01; ***P < 0.001; ****P < 0.0001; ns, no statistical significance. (TIF) [file pntd.0012163.s002.tif]

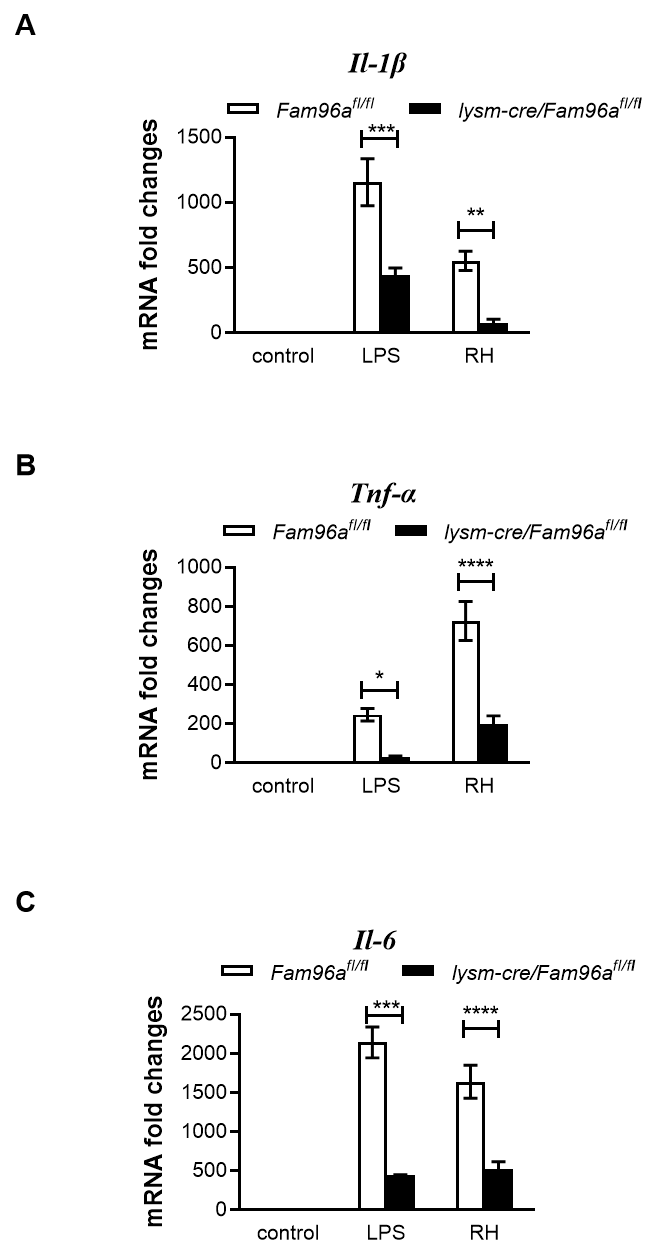

Supplement: S2 Fig — Peritoneal macrophages isolated from Fam96afl/fl and Lysm-cre/Fam96afl/fl mice were stimulated with LPS (100 ng/mL) or T. gondii tachyzoite RH (MOI = 2) for 4 h in vitro. The mRNA expression levels of (A) Il-1β, (B) Tnf-α, and (C) Il-6 were then evaluated by qRT-PCR. **P < 0.01; ***P < 0.001; (TIF) [file pntd.0012163.s003.tif]

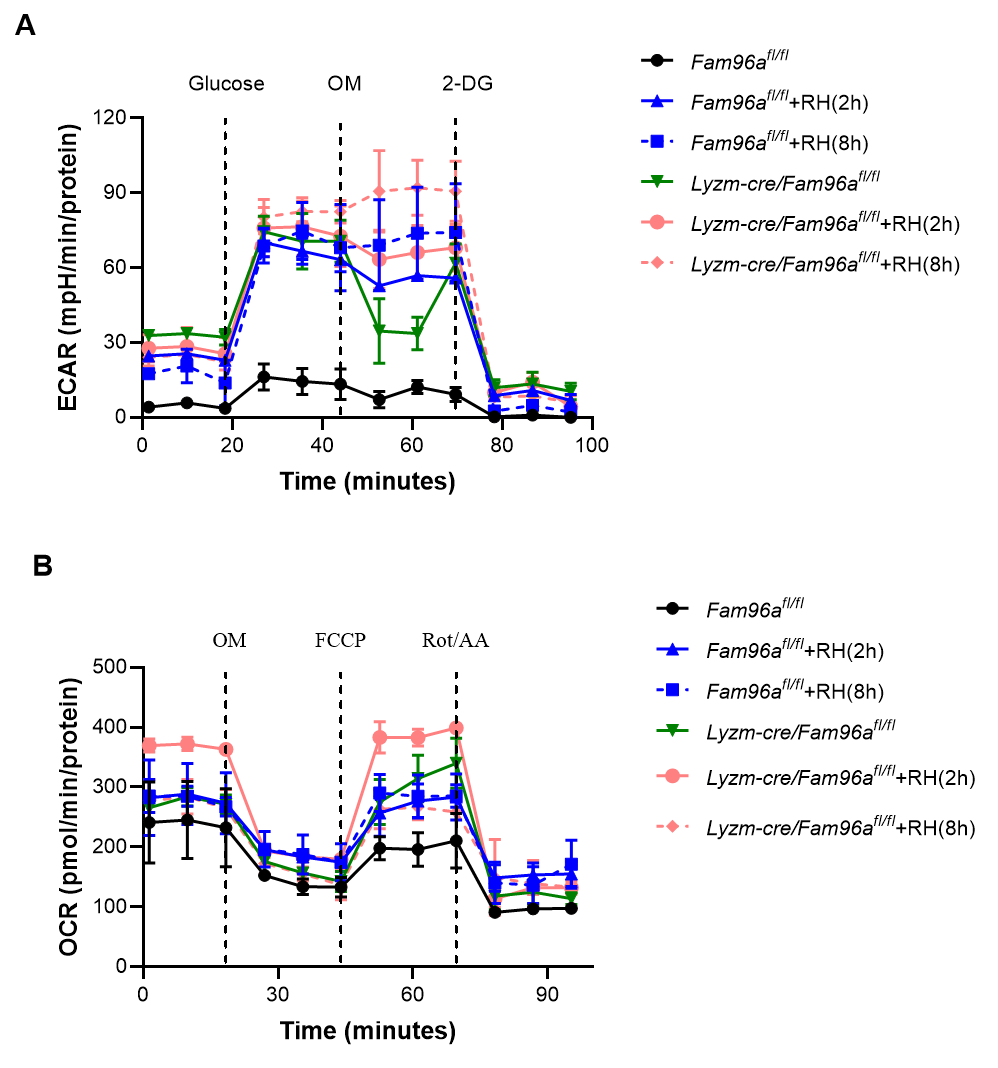

Supplement: S3 Fig — (A) the three individual ECAR graphs seen in the main Fig 4A were merged in this graph. (B) the three individual OCR graphs seen in the main Fig 4B were merged in this graph. (TIF) [file pntd.0012163.s004.tif]
